# Do Left and Right have Temporal Meaning?

**DOI:** 10.3389/fpsyg.2012.00433

**Published:** 2012-11-02

**Authors:** Kevin Ezra Moore

**Affiliations:** ^1^Department of Linguistics and Language Development, San José State UniversitySan José, CA, USA

De Sousa's paper makes a valuable contribution to our understanding of spatial representations of temporal phenomena, and helps set the stage for further advancement.

The question of what the left-to-right (LR) or right-to-left (RL) order of card arrangements means is a pressing one. To what extent are the non-linguistic demonstrations such as the placement of cards analogous to linguistic expressions that use words like *follow* to depict times/events in one-dimensional order? Perhaps LR, RL, up-down, and down-up all represent the same temporal concept. That is, perhaps the meaningful characteristics of the observed configurations are (i) the one-dimensional directional order (but not the orientation), and (ii) the fact that ego is not part of the array.

One way of interpreting the results of the card-arrangement task would be that they are evidence of a mental routine for creating and interpreting one-dimensional order in a set of physical objects. But what is the relationship between the order of the cards and the subject's understanding of the successive stages pictured in the cards? What if subjects were asked to put items in order according to size? We might see a strategy for putting things in order in which the LR or RL direction did not reveal anything about the conceptual structure of size. What if subjects were asked to arrange “sequence” cards in temporal order but to put down the card representing last stage first (e.g., the empty banana peel first)? Will subjects use the same LR or RL order as they did when putting down the first stage first? The results of an experiment like this might tell us if the LR or RL direction has to do with the act of laying out the cards in sequence or with the sequence pictured on the cards. (As de Sousa points out, the card task could have influenced the results of the time-points task, so I do not discuss the time-points task here.)

If the direction in which de Sousa's subjects laid out cards is ultimately based on direction of reading/writing, the simplest characterization is that the subjects used a well-learned routine of comprehending or producing order and applied it to a new situation (De Sousa makes a similar suggestion on pages 13–14. Cf. Ramscar et al., 2010, p. 7). If so, the results of the card-arranging task do not show a spatial conception of time so much as a previously learned spatial/temporal strategy applied to a new spatial/temporal situation. That is, knowledge and experience of reading/writing, which has spatial and temporal dimensions, influences card-arranging, which has the same spatial and temporal dimensions. This observation also applies to other work on reading/writing direction and card-arranging, and this discussion is also relevant to work that characterizes motion metaphors of time (e.g., *Christmas is coming*) as conceiving of time in terms of space, since motion involves time. That is, these data (non-linguistic and linguistic) are evidence for concepts that involve both space and time, which, strictly speaking, is not the same as *a spatial conception of time*.

We clearly benefit from studying how people take their understanding of sequence from reading/writing and apply it to other instances of sequence. A next step will be to determine which aspects of the LR or RL configurations are meaningful and what they mean. Núñez and Sweetser (2006) could say that a gesture to the front meant “in front of ego” partly because the gesture correlated with a word for front. Of course it is part of the value of work like de Sousa's that the data are largely independent of language, but as we continue we will want to know how the conceptual structure revealed by data like those in de Sousa's paper relates to the conceptual structures that people have in mind when they talk about sequence.
